# The breath shape controls intonation of mouse vocalizations

**DOI:** 10.1101/2023.10.16.562597

**Published:** 2023-10-17

**Authors:** Alastair MacDonald, Kevin Yackle

**Affiliations:** 1 Department of Physiology, University of California-San Francisco, San Francisco, CA 94143

## Abstract

Intonation in speech is the control of vocal pitch to layer expressive meaning to communication, like increasing pitch to indicate a question. Also, stereotyped patterns of pitch are used to create distinct “words”, like the ten sounds in the murine lexicon. A basic tone is created by exhalation through a constricted laryngeal voice box, and it is thought that more complex utterances are produced solely by dynamic changes in laryngeal tension. But perhaps, the shifting pitch also results from altering the power of exhalation. Consistent with the latter model, we describe that intonation in many adult murine vocalizations follows deviations in exhalation and that the brainstem vocalization central pattern generator, the iRO, can create this breath pattern. Consequently, ectopic activation of the iRO not only induces phonation, but also the pitch patterns that compose most of the vocalizations in the murine lexicon. These results reveal a novel brainstem mechanism for intonation.

## Introduction:

Modulation of the frequency of produced sound, perceived as pitch, creates meaning within words or phrases through intonation (Prieto et al., 2015). For example, in English, an increasing pitch is used to indicate a question or stress importance and a decreasing pitch communicates a declaration. Additionally, the concatenation of specialized sounds with variation in pitch, like syllables, composes the diverse repertoire of words (Poeppel et al. 2020). Two key pieces of the phonation system are the larynx (the “voice-box”) and the breathing muscles (Berke et al. 2009, Laplange et al., 2018). Succinctly, the breathing muscles drive airflow through a narrowed larynx to produce a basic vocalization (Finck et al., 2009). The speed of the airflow through the larynx dictates the fundamental frequency of the tone, so changes in either the forcefulness of the breath exhalation or the extent of laryngeal closure can both, presumably, alter the pitch (Kelm-Nelson et al., 2018, Herbst C.T., 2016, Mahrt et al., 2016). While control of the size of the laryngeal opening is well established as a mechanism to regulate the dynamic changes in pitch for human to rats and mouse vocalizations (Poeppel et al. 2020, Johnson et al., 2010, Riede et al., 2017), the contribution of exhalation itself remains to be carefully defined. In fact, it is presumed that the forcefulness of expiration only modulates the vocal amplitude or loudness (Riede T., 2011, Riede T., 2013) This perception stems from the airflow of the rodent breath not strongly predicting the pitch. Yet paradoxically, an injection of air below the larynx to enhance airflow increases pitch (Riede T., 2011). This incongruity even extends into songbirds, a leading vocalization model system (Suthers et al., 2002, Schmidt et al., 2014, Plummer et al., 2008, Goller et al., 2004). Here, we seek to resolve this inconsistency by taking advantage of the experimental, behavioral, and genetic approaches in the mouse (Yackle K., 2023). If two independent variables are used to alter pitch, like the larynx and the breath airflow, then the interplay would enhance the ability to produce a diverse repertoire of sounds and thereby enable a broader lexicon.

The medullary brainstem possesses at least two means that might account for the two control points proposed above, laryngeal diameter and exhalation strength. First, direct modulation of laryngeal premotor and motor neurons in the retroambiguus (RAm) modulates the size of the laryngeal opening (Kelm-Nelson et al., 2018, Hage S.R., 2009). And second, the vocalization central pattern generator we recently described, called the intermediate Reticular Oscillator (iRO), induces coordinated changes in the expiratory airflow and laryngeal closure (Wei et al., 2022). For example, during neonatal cries, the iRO oscillates exhalation strength and larynx activity to time the syllable sounds. Thus, the RAm provides a mechanism to modulate pitch by controlling laryngeal diameter independently from the iRO altering the tone by dictating the extent of the breath expiratory airflow. While the contribution of RAm in adult phonation has been established (Jürgens U., 2002), the role of the iRO remains undefined.

Here, we describe the coordinated changes in breath airflow and pitch in the ten vocalizations of the adult murine lexicon (Grimsley et al., 2011). We describe that the modulation of pitch for the different vocalizations either correlates or anticorrelates with the changes in exhalation. These results support a model in which two independent mechanisms involving changes in laryngeal opening or airflow control tone. Using anatomical, molecular, and functional approaches, we demonstrate that the iRO vocal central pattern generator drives changes in breath expiratory airflow to pattern pitch and produce seven of the ten vocalizations in the endogenous lexicon. These data resolve the prior paradoxical role of exhalation and show it can directly control pitch. Additionally, we establish the iRO as a mechanistic basis for intonation. And lasty, these results generalize the crucial role of the iRO in phonation across developmental stages, and we presume across species.

## Results:

### Vocalizations are produced by a program coupled to breathing.

**Figure 1.**
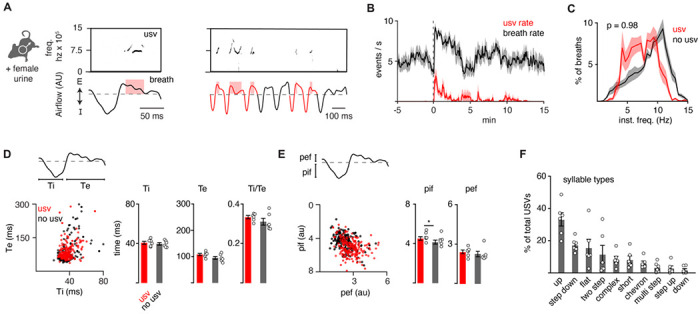
The full repertoire of vocalizations occurs within a normal appearing breath. **A**, Male mice exposed to female urine produce ultrasonic vocalizations (USV) at about 75 kilohertz (top) that coincide with the expiratory airflow (E, arbitrary units) of the breath cycle (bottom). Red box indicates the length of the USV. A bout of vocalizations contains breaths with USVs (red) interspersed among sniff breaths (black). **B**, Rates of breathing (black) and USV production (red). Exposure to female urine at time 0, n=6 mice. **C**, Histogram of the instantaneous frequency of breaths with and without USVs from n=6 animals. p-value 0.98; two-way ANOVA. **D**, Scatter plot of the inspiratory (Ti) and expiratory time (Te) for USV (red) and non-USV (black) breaths from n=1 representative animal. Right, bar graph of mean ± SEM of Ti, Te, and the ratio for n=6. Each dot is the mean from each animal. p-values 0.40, 0.18, and 0.25; paired t-test. **E**, The breath peak inspiratory (pif) and expiratory (pef) airflow represented as in **D**. p-values 0.01, 0.27; paired t-test. **F**, Bar graph (mean ±SEM) of the percent of total USVs for each type from n=6 mice. Each dot is the mean from each animal.

It is possible that the ten murine ultrasonic vocalizations (USVs) defined by unique pitch patterns (Grimsley et al., 2011) are formed by distinct breaths or as substructures nested in a common breath. Prior work has suggested the latter (Sirotin et al., 2014). To expand upon this, we simultaneously measured breathing and USVs by customizing the lid of a whole-body plethysmography chamber to accommodate a microphone. Male mice in the chamber were exposed to fresh female urine and robustly sniffed and vocalized for the first 5-10 minutes of the recording at a peak rate of about 4 events per second (n = 6) (Fig. 1A–B). A vocalization was classified as a narrow-band sound in the 40-120 kHz ultrasonic frequency range during a single breath (Fig. 1A). The rate of vocalization breaths was typically between 5-10 Hz (Fig. 1C) and mostly occurred during episodes of rapid sniffs (~10 Hz) (Fig. 1A and B), as previously reported (Sirotin et al., 2014, Castellucci et al., 2018) When compared to neighboring breaths, vocalization breaths had slightly larger inspiratory and perhaps expiratory airflow despite similar durations of each phase (Fig. 1D and E). These data reveal that a vocalization breath appears mostly like a normal breath, but with the addition of a nested sound pattern. This led us to hypothesize that a distinct sub-program is activated within a breath to generate a vocalization.

The adult murine lexicon is composed of at least 10 USV types that are defined by different, but stereotyped patterns of pitch. One breath can contain multiple syllables, which we define as a continuous USV event within a breath (Fig. 1F and S2–3). A pre-trained convolutional neural network (CNN) was used to classify USVs into different types based on changes in pitch (Fonseca et al., 2021) and the on- and offset of each vocalization was overlayed upon the corresponding breath airflow (Fig. S1–3). Vocalizations began and ended throughout expiration (Fig. S1), and the most common tended to start near the onset of exhalation and ended shortly there-after (like the up frequency modulated, step down, flat, and short types) (Fig. S2). Vocalizations with more intricate changes in pitch had more variable times of on- and offset (like complex, chevron, two step, multi, step up, down frequency modulated) (Fig. S3). And lastly, when the vocalizations occurred late in expiration, the duration of this breath phase was prolonged (Fig. S4). The bias of USV timing by the breath, combined with the USV modulation of breath length demonstrate these programs are independent but reciprocally coupled.

### Two mechanisms create the changes in pitch pattern.

**Figure 2.**
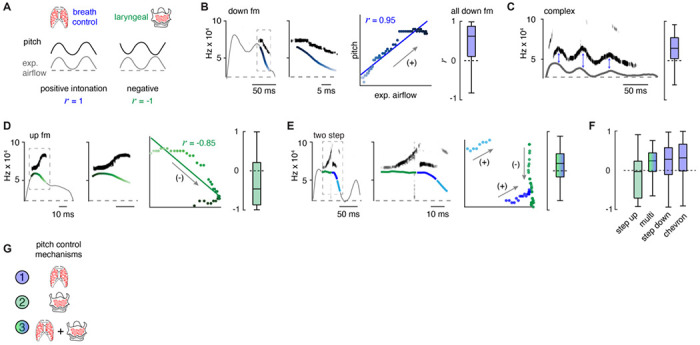
The ten types of USVs are produced by at least two mechanisms that modulate airflow. **A**, Two models to control the USV pitch by changing the speed of airflow through the larynx. Left, modulation in expiratory airflow drives the change in pitch (positive intonation, blue). Right, the change in pitch anti-correlates with airflow suggests closed larynx produces sound (negative intonation, green). **B**, Left, example of the expiratory airflow and pitch for a down frequency modulated (fm) USV. Middle, magnification of airflow and sound. The scale of airflow is not displayed. The time of breath airflow from expiration onset during the USV is color coded blue to white. Note, the change in pitch mirrors airflow, consistent with the ‘breath modulation’ mechanism (annotated as “+”). Scatter plot of instantaneous expiratory airflow and pitch for a single USV and the correlation (line, *r*). Box and whisker plot of n=40 down fm correlation coefficients (*r*). **C**, Representative expiratory airflow and pitch and box and whisker plot of all *r* values for complex (n=165) vocalizations. **D**, Up frequency modulated (fm) vocalization represented as in **B** (n=589). Note, the change in pitch opposes the airflow, consistent with the ‘laryngeal closure’ mechanism. (annotated as “-”). **E**, Two step vocalization represented as in **B** (n=61). The airflow for each unique USV element is uniquely color coded as green, blue, or purple. Note, the change in pitch for two components correlates and one anticorrelates. This is consistent with both mechanisms being sequentially used. Annotated as mixed blue and green box and whisker plot. **F**, Box and whisker plot of correlation coefficients (*r*) for step up (n=340), multi (n=58), step down (n=293), and chevron (n=99). **G**, Model schematic of the mechanisms used to control pitch for the various USV types.

Fluctuations in airflow through the larynx produce changes in the sound’s pitch. For example, augmenting airflow through the explanted rodent larynx increases pitch (Mahrt et al., 2016). We proposed two potential models that would explain how the laryngeal airflow is modulated to form the distinct USV types in the murine lexicon: one based on the strength of exhalation pushing air through the larynx, and another based on the diameter of the laryngeal opening (Fig. 2A). According to the first model which we term positive intonation, if the pitch changes mirror the modulation of the breath expiratory airflow, the plethysmography airflow and pitch will simultaneously fluctuate (Fig. 2A, left). In the opponent model, negative intonation, a narrowed larynx used to increase pitch would impede the overall expiratory airflow such that pitch is anticorrelated with plethysmography airflow (Fig. 2A, right). Note, these models can form similar airflow patterns, but predict opposite relationships to pitch.

We assessed each model by calculating the correlation coefficient (*r*) between instantaneous expiratory airflow and the corresponding USV fundamental frequency. Down or up frequency modulated USVs were positively or negatively correlated, respectively (median *r* = 0.62 and −0.46, Fig. 2B and D). These simple USVs reflected the two proposed mechanisms to alter pitch, positive and negative intonation (Fig. 2A). Six of the other ten USVs types had positively shifted intonation, the chevron, complex, step down, multi, and two step types (median *r* = 0.32, 0.31, 0.28, 0.24, and 0.19 respectively), and many of the step up were negatively biased (median *r* = −0.03) (Fig. 2C, E, F). For many of these USV types, it appeared that a portion of the USV pattern correlated with the expiratory airflow, while the other part(s) were un- or anticorrelated (e.g., the two step, Fig. 2E). This suggests that the pitch is patterned by switching between positive and negative intonation mechanisms within the breath (Fig. 2G). The remaining two USV types (flat and short) had different breath shapes, which resulted in a wide range of *r* values (Fig. 4). In summary, all these results support the hypothesis that a vocalization pattern generator must integrate with and even control the breath airflow as a key mechanism to produce various USV types in the murine lexicon (Fig. 2G).

### The iRO resides within the adult brainstem phonation circuit.

Murine vocalizations are innate and stereotyped (Fig. S1 and 2) which predicts they are generated by a vocal central pattern generator (CPG). The similarities between the positively correlated USV types and the neonatal cry vocalizations produced by a vocal CPG known as the intermediate Reticular Oscillator or iRO (Wei et al., 2022) suggests the iRO is involved in generating adult USVs. However, the iRO has yet to be identified in adult mice.

**Figure 3.**
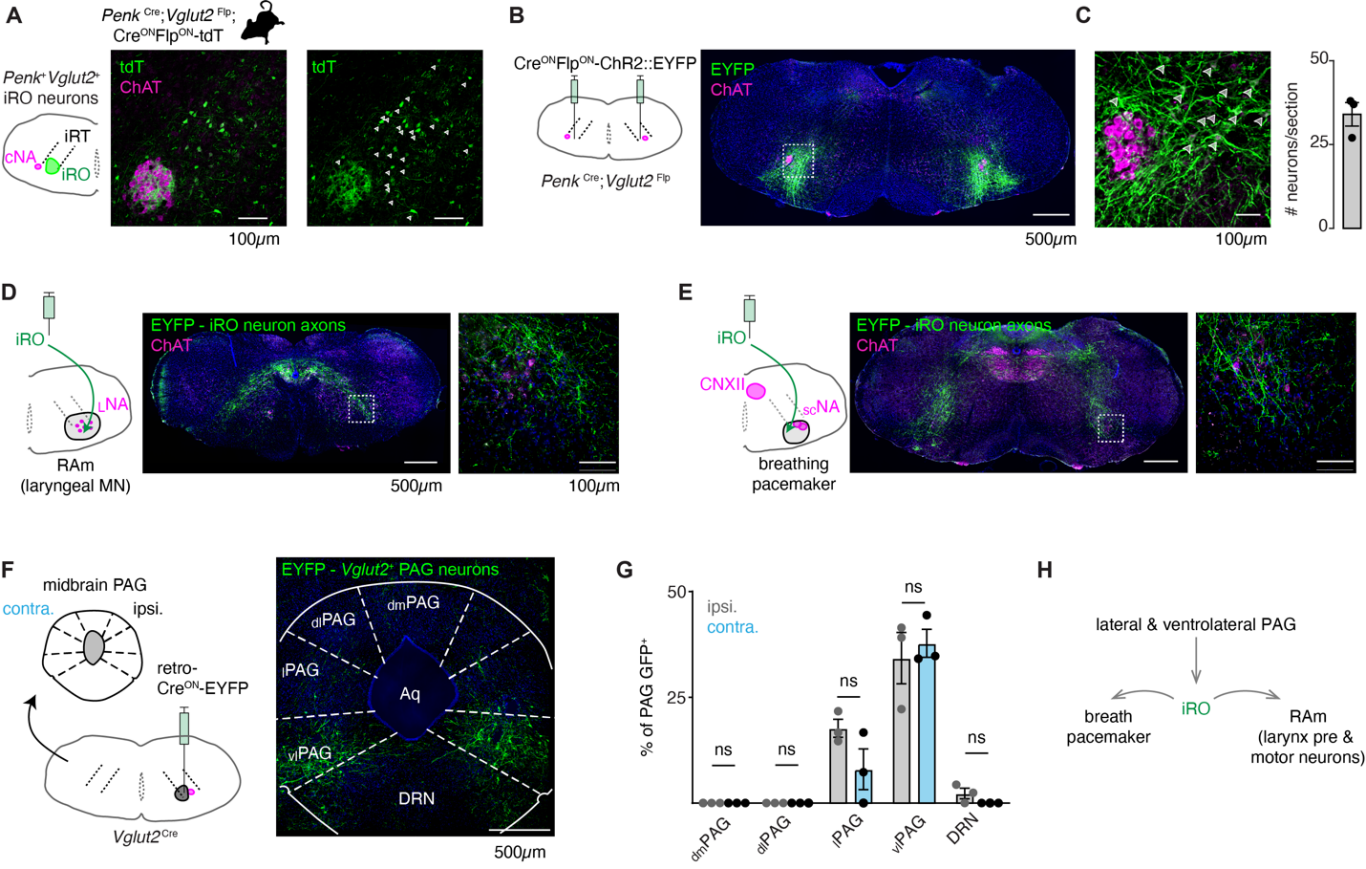
Anatomically and molecularly defined iRO neurons form a brainstem phonatory circuit. **A**, Labeling of *Penk*+*Vglut2*+ neurons in the iRO anatomical region in adult *Penk*-Cre; *Vglut2*-Flp;Ai65 mice (Cre^ON^Flp^ON^-tdTomato) (observed in n=5 mice). The iRO region is defined as medial to the compact nucleus ambiguus (cNA, ChAT +) in the ventral intermediate Reticular Tract (iRT). Note, the cNA is filled with tdTomato labeled axons. Cell bodies marked with arrowhead. **B**, Bilateral stereotaxic injection of AAV Cre^ON^Flp^ON^-ChR2::EYFP into the iRO anatomical region of *Penk*-Cre; *Vglut2*-Flp adult mice. **C**, Magnified boxed region in **B**. Arrowheads label neuron soma quantified right (n=3). **D**, Axons of EYFP expressing iRO neurons from **B** in the retroambiguus (RAm) where laryngeal motor neurons are located. **E**, Axons of EYFP expressing iRO neurons from **B** in the breathing pacemaker. **F**, Unilateral retrograde AAV Cre^ON^-EYP (AAVrg) stereotaxic injection into the iRO region in *Vglut2*-Cre adults (n=3). Glutamatergic neurons were identified in the contralateral (contra.) and ipsilateral (ipsi.) midbrain periaqueductal gray (PAG). Anatomical regions of the PAG: dorsomedial (dm), dorsolateral (dl), lateral (l), ventrolateral (vl) nearby to the dorsal raphe nucleus (DRN) and surrounding the cerebral aqueduct (Aq). **G**, Quantification of glutamatergic PAG neurons in each region demarcated in **F**, ns = not statistically significant; two-way ANOVA with Sidka’s post-hoc test. **H**, Model schematic of the iRO as a central component of the brainstem phonation circuit to convert a vocalization “go” cue from the PAG into a motor pattern.

The iRO is molecularly defined in the neonate by the co-expression of *Preproenkephalin* (*Penk*) and *Vesicular glutamate transporter 2* (*Vglut2*) and is anatomically localized to the medullary ventral intermediate Reticular Formation (iRT) directly medial to the compact nucleus ambiguus^17,19^ (Wei et al., 2022). This general region has been dubbed the Post inspiratory Complex (PiCo) given its involvement in post-inspiration, including behaviors like swallowing (*Anderson et al., 2014,* Huff et al., 2023). We determined that the iRO molecular and anatomical features exists in adults in two ways. First, we generated triple transgenic mice that label *Penk*^+^*Vglut2*^+^ neurons and the derived lineages with tdTomato (*Penk*-Cre; *Vglut2*-Flp; Ai65) (Fig. 3A). And second, we stereotaxically injected the iRO region of *Penk*-Cre; *Vglut2*-Flp mice with a Cre and Flp dependent reporter adeno-associated virus (AAV Cre^ON^Flp^ON^-ChR2::YFP) (Fig. 3B–C). Consistent with the definition of the iRO in neonatal mice, tdTomato+ and YFP+ *Penk*^+^*Vglut2*^+^ neurons were found in the iRT adjacent to the compact nucleus ambiguus (Fig. 3A–C). These results demonstrate that the ventrolateral medulla of adult mice contains neurons with the molecular and anatomical identity of the iRO.

Neonatal iRO neurons are presynaptic to the kernel of the breathing, the pacemaker for inspiration (preBötzinger Complex, preBötC) (Smith et al., 1991) and premotor to multiple laryngeal and tongue muscles. We traced the YFP+ axons of *Penk*^+^*Vglut2*^+^ neurons (*Penk*-Cre;*Vglut2*-Flp and AAV Cre^ON^Flp^ON^-ChR2::YFP) and found they elaborated within the nucleus ambiguus (NA) and retroambiguus (RAm) where laryngeal premotor and motor neurons localize (Fig. 3A, D), the breathing pacemaker (Fig. 3E), and the hypoglossal (tongue) motor nucleus (Fig. 3E). The projection patterns of these *Penk*^+^*Vglut2*^+^ neurons provide additional evidence that these adult neurons maintain the same connectivity properties as the neonatal iRO neurons, indicating they can control the key elements for vocalization: the breath airflow and larynx.

In adult mice, vocalizations have been triggered by activation of the midbrain periaqueductal gray (PAG), namely glutamatergic neurons in the ventrolateral subregion (Michael et al., 2020, Chen et al., 2021, Tschida et al., 2019). To assess if the iRO region is positioned downstream of the ventrolateral PAG, we unilaterally injected *Vglut2*-Cre mice with a Cre^ON^-ChR2::YFP expressing retrograde traveling AAV (AAVrg) (Fig. 3F). Among the labeled brain regions, we found YFP+ neurons selectively in the phonation region of the midbrain PAG. To our surprise, neurons from the ipsi- and contralateral PAG projected to the iRO region in nearly equal numbers (Fig. 3G). These molecular, anatomical, and neural morphology characterizations reveal that the iRO exists in adults and is embedded within the brainstem phonation network (PAG → iRO → the preBötC, NA, RAm, hypoglossal) (Fig. 3H).

### Ectopic activation of the putative iRO induced vocalization.

**Figure 4.**
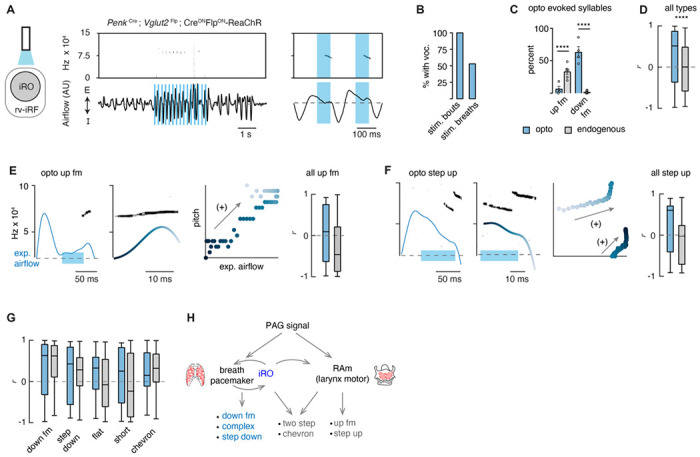
Ectopic activation of the iRO evokes airflow correlated USV types and switches the relationship of the anti-correlated types. **A**, Optogenetic activation of the iRO region in *Penk*-Cre;*Vglut2*-Flp;Cre^ON^Flp^ON^-ReaChR mice evokes USVs (blue box, 5 Hz stimulation). USVs occur during, or shortly after laser onset. **B**, Percentage of stimulation bouts containing at least one USV and the percentage of breaths within the stimulation window containing a USV. **C**, Percentage of optogenetically evoked (blue) or endogenously occurring (gray) syllables that are up fm of down fm. **** p-value< 0.001; two-way ANOVA with Sidak’s post-hoc test, p > 0.05 for all other types. **D**, Box and whisker plot of the correlation coefficient between breathing airflow and pitch (*r*) for all opto evoked (n=395) and endogenous (n=1850) USVs from n=4 opto and n=6 endogenous mice. **** p-value < 0.001; Mann-Whitney test. **E,** Left, example of the expiratory airflow and pitch for an optogenetically evoked up fm USV. Middle, magnification of airflow and sound. Time of breath airflow during the USV is color coded blue to white. Scatter plot of instantaneous expiratory airflow and pitch for the single USV. Compare to endogenous up fm USV in Fig. 2D. Right, box and whisker plot of correlation coefficients (*r*) for each optogenetically evoked and endogenous up fm USV (n=15 vs. 589). **F**, Step up USV as in **E**. box and whisker plot of correlation coefficients (*r*) for each optogenetically evoked and endogenous step up USV (n=15 vs. 40). **G**, *r* value box and whisker plots for the remaining optogenetically evoked USV types (down fm n=242 vs. 40, step down n=38 vs. 293, flat n=34 vs. 337, short n=27 vs. 168, and chevron n=10 vs. 99 from n=4 opto and 6 endogenous mice. **E-G**, two way ANOVA with Sidak’s post-hoc test for two way comparisons was used; all p-values >0.05. **H**, Schematic illustrating the two mechanisms to pattern the USV pitch. Left, the reciprocal connection between the iRO and breathing pacemaker patterns the USVs with a positive correlation between pitch and airflow. Right, the retroambiguus (RAm) control of the larynx dictates the anti-correlated USV types. Middle, the combination of these two mechanisms within a single breath create additional USV patterns.

If these labeled *Penk*^+^*Vglut2*^+^ neurons are indeed the iRO, we anticipated that ectopic activation would induce vocalization. We tested this in two ways. First, we generated *Penk*-Cre; *Vglut2*-Flp;Cre^ON^Flp^ON^-ReaChR triple transgenic mice which express the red-shifted Channel Rhodopsin in *Penk*^+^;*Vglut2*^+^ neurons and the derived lineage (ReaChR mice) and second, we stereotaxically injected the AAV Cre^ON^Flp^ON^-Channel Rhodopsin2::YFP (ChR2) into the iRO region of *Penk*-Cre;*Vglut2*-Flp mice. In both instances we implanted optic fibers above the iRO bilaterally to further localize neural activation (Fig. 4A and S5A). In both experimental regimes, ectopic light activation of the *Penk*^+^*Vglut2*^+^ neurons induced bouts of vocalizations where the breathing rate was entrained by the frequency of stimulation (Fig. 4A, S5A, S5I). Most bouts and the breaths within contained vocalizations (Fig. 4B and S5B), and the amplitudes of all elicited breaths were significantly increased (Fig. S5J). Some AAV-ChR2 mice showed broad band vocalizations, while others did not vocalize, likely due to incomplete labeling (n=5/9). Additionally, the ReaChR animals without vocalizations were found to have “off target” optic fiber implants (n=2/6). Taken together, these data are consistent with the notion that the iRO is sufficient to induce phonation via control of both breath airflow and laryngeal opening, just as it does in neonatal cries.

To demonstrate the specialization of the iRO neurons for vocalization and the inability of modulated breathing alone to elicit USVs, we performed several additional control experiments. First, to ensure that just stimulation of breathing is insufficient to elicit vocalization, we optogenetically excited the glutamatergic preBötC neurons (*Vglut2*-Cre with AAV Cre^ON^-ChR2). Indeed, we found that, although breathing sped up, optogenetic stimulation never elicited vocalizations (Fig. S5C, H, I). And second, to determine if the ability to elicit vocalizations was generalizable to other neural types in the iRO anatomical region, we activated *Penk*^+^, *μ-opioid receptor*^+^*Vglut2*^+^, *Tachykinin 1*^+^, and *Vesicular GABA transporter*^+^ neurons and found that vocalizations were never induced upon light stimulation, although breathing was altered in various ways (Fig. S5). In summary, these data functionally demonstrate the existence of *Penk*^+^*Vglut2*^+^ iRO neurons in adult mice and their ability to create vocalizations by modulating both breathing and presumably the larynx.

### Excitation of the iRO evoked nearly the entire murine lexicon.

Above, we described that one mechanism for generating the different patterns of vocalizations was via the modulation of the breath airflow (positive intonation). Once again, this was defined as a positive correlation between expiratory airflow and pitch (Fig. 2). We hypothesized that this property stems from the iRO’s capacity to control breathing, and so we made the following predictions: 1) that the USVs evoked after stimulation would be biased to those with an endogenous positive correlation between airflow and pitch (like the down fm and step down), and 2) that any of the elicited USV types would be transformed to become more positively correlated.

We classified the evoked iRO vocalizations (*Penk*-Cre;*Vglut2*-Flp;Cre^ON^Flp^ON^-ReaChR ) with the CNN, and to our surprise, seven of the ten types of endogenous USVs were induced upon activation of the iRO (Fig. 4). The most abundant elicited USV was the down fm which, in the endogenous dataset, had the strongest positive intonation (Fig 4C and Fig. 2). Conversely, the USV with the strongest negative intonation was rarely found, up fm. These results are striking since the down fm is the least common endogenous USV and up fm is the most common (Fig. 2). These results are consistent with the first prediction where the optically evoked USV types were biased towards those with endogenous positive intonation. Beyond this, all the ectopic USVs were transformed to positively associate airflow and pitch, even when the counterpart endogenous USV was un- or anticorrelated (e.g., up fm and step up USVs) (Fig. 4E–F). This aligns with the second prediction. These data demonstrate that the iRO is sufficient to pattern nearly all USV types, and that the pitch of the induced vocalizations tightly follows the breathing airflow.

## Discussion:

Here we propose that the intonation that establishes the diversity of the adult murine lexicon is explained by two mechanisms, the modulation of the breath waveform and presumably the size of the laryngeal opening. First, we describe that unique vocalization types have characteristic fluctuations in the expiratory airflow, whereby some changes in pitch are strongly correlated with airflow while others are anticorrelated. These two mechanisms can even be used in the same breath to produce complex changes in pitch. To our surprise, six of the ten USV types primary used the positive intonation mechanism. These data support a novel and key role for the breathing system in the production of various types of vocalizations. Second, we show that the vocalization central pattern generator, the iRO, is sufficient to induce most of the endogenous USVs types via the modulation of the breath airflow. In contrast to the natural lexicon, the pitch of the evoked USVs is explained by positive intonation. These data imply that the iRO can produce the mechanism to pattern positive intonation, and thereby suggests that negative intonation derives from a separate neuronal component of the phonatory system. We propose these two mechanisms can be used independently or in conjunction to generate the diverse repertoire of vocalizations (Fig. 4H).

### The iRO likely patterns intonation for endogenous phonation.

The description of the iRO within the adult neural circuit for phonation suggests a key role in patterning the endogenous adult vocalizations. In this case, we propose that the upstream periaqueductal gray input would “turn-on” the iRO which then co-opts the breathing pacemaker and coordinates its modulation with laryngeal activity to produce and pattern the coordinated changes in breath airflow and vocal pitch (positive intonation). The iRO can do this since it is presynaptic to both the breathing pacemaker and the laryngeal motor neurons. In this case, the brief re-activation of inspiratory muscles would slow ongoing expiration, enabling bi-direction changes in airflow, and thus pitch. This type of modulation has been demonstrated in neonatal cries (Wei et al., 2022). An important next step will be to validate this supposition by correlating measurements of breathing muscle activity with pitch. Also, future studies should explore the need of the iRO in adult phonation, as anticipated from its necessary role in neonatal vocalization. None-the-less, the presence of the iRO across developmental stages implies a conserved role in innate vocalizations within the mouse and perhaps across the animal kingdom, where vocalization central pattern generators have been hypothesized and even identified in species from fish to birds to primates (Zhang et al., 2020, Chagnaud et al., 2011, Hage S.R., 2009, Kelley et al., 2020).

### The iRO can autonomously produce multiple vocalization patterns.

A surprising finding is that ectopic activation of the iRO produces seven of the ten vocalization types within the murine lexicon. How might this occur? One possibility is that the iRO has multiple modes which can each produce a different pattern of activity. Such a phenomena has been demonstrated in other central pattern generating systems like the crustacean stomatogastric ganglia (Marder et al., 2001, Marder E., 2012). A more likely option is that additional mechanisms of vocal modulation are layered upon a basic pattern produced by the iRO. For example, other regions with direct control of the laryngeal motor neurons within RAm would add complexity to the vocalization induced by the iRO, akin to how vocal control by the human laryngeal motor cortex is perceived (Fig. 4H) (Dichter et al., 2018, Silva et al., 2022). Here we propose that perhaps just two mechanisms (breath airflow and laryngeal opening) account for the intricacy of the murine sounds produced, and the layering of these enables a basic pitch structure within a breath to become sophisticated.

### The control of breathing airflow is a novel biomechanical mechanism for intonation.

Intonation is a key aspect of communication, whereby the same word or phrase could be used as a question or a statement simply by different fluctuations in pitch. Our findings describe a novel biophysical mechanism for intonation and a cellular basis. Now, the iRO or the direct modulation of breathing can serve as a starting point to map higher level components of brain-wide vocalization circuits that structure additional subliminal layers of perception in speech.

## Resource Availability

### Lead contact

Further information and requests for resources and reagents should be directed to and will be fulfilled by Kevin Yackle (kevin.yackle@ucsf.edu).

### Materials availability

This study did not generate new unique reagents.

### Data and code availability

- All reported data collected in this study will be shared by the lead author upon request.
- All original code has been deposited at Github. DOIs are listed in the key resources table.
- Any additional information required to reanalyze the data reported in this paper is available from the lead contact upon request.

## Experimental model and subject details

*Vglut2*^FlpO^, *Penk*^Cre^, *Tac1*^Cre^, *Oprm1*^Cre^, *Vgat*^Cre^, Ai65 and LSL-FSF-ReaChR have been described. Mice were obtained from Jackson laboratories and bred in house at the UCSF Laboratory Animal Research Center. Mice were housed in groups of 2-5 unless otherwise stated under a 12:12 light-dark cycle with *ad libitum* access to chow and water. All animal experiments were performed in accordance with national and Institutional Animal Care and Use Committee - University of California San Francisco guidelines with standard precautions to minimize animal stress and the number of animals used in each experiment.

### Recombinant viruses

All viral procedures followed the Biosafety Guidelines approved by the University of California, San Francisco (UCSF) Institutional Animal Care and Use Program (IACUC) and Institutional Biosafety Committee (IBC). The viruses used in experiments were AAV5-hSyn-Con/Fon-hChR2(H134R)-EYFP (55645-AAV5, Addgene, 1.8x1013 vg/ml), AAV5-EF1a-DIO-hChR2(H134R)-EYFP-WPRE-HGHpA (20298-AAV5, Addgene, 1x1013 vg/ml), AAVrg-EF1a-DIO-hChR2(H134R)-EYFP-WPRE-HGHpA (20298-AAVrg, Addgene, 2.1x1013 vg/ml).

## Methods details

### Endogenous USV and breathing recording

Male *Vglut2*^FlpO^;*Penk*^Cre^ mice (aged 8-16 weeks) were individually housed and habituated to experimenter handling and a plethysmography chamber for >4 days. On the test day the mice were placed in a clean cage base with a female mouse for 5 minutes and then moved to a plethysmography chamber. The chamber was modified to accommodate a microphone to record vocalizations (CM16/CMPA, Avisoft Bioacoustics) and the airflow in the chamber was measured by a spirometer (FE141, AD instruments). Both data streams were acquired through a DAQ board (PCI-6251, National Instruments) and written to disk for offline analysis. Sound was acquired at 400 kHz and airflow at 1 kHz. After a 20-minute habituation period, mice had airflow and sound recorded for 5 minutes before a cotton bud soaked in fresh urine was placed in the chamber and sound and breathing were recorded for a further 15 minutes. Urine was collected the day of the experiment from a group of 5 female mice temporarily housed in a custom-made wire-bottom cage.

The recordings were run through VocalMat (Fonseca et al 2021) for USV detection and only mice that produced >50 USVs in response to the stimulus were included for further analysis (5/13 mice). Airflow recordings were imported to MATLAB, high pass filtered (2Hz) and smoothed. Breaths were taken from the first 200s following urine presentation and features (Ti, Te, Pif, Pef, instantaneous frequency) were computed from segmented breaths as previously described (Bachmustky et al 2021). USV start and end times from VocalMat were used to identify which breaths contained USVs and calculate timing metrics (relative onset and offset from expiration onset and the same values normalized to expiratory duration). VocalMat was also used to identify the types of USV which were manually checked and corrected if necessary. For analysis of the relationship between airflow and frequency a multitaper spectrogram was computed using code modified from USVseg (Tachibana et al 2020) and then the frequency bin with the greatest power was taken from each time bin to create a vector of the peak frequency. The correlation coefficient of this peak frequency vector and the expiratory airflow at the time stamps identified by VocalMat was then calculated for each identified USV.

### Virus injection, fiber implantation and optogenetics

Surgery was conducted with sterile tools and aseptic technique. Mice were first anaesthetized with isofluorane (4%), the hair overlaying the scalp was shaved and mice were placed in the stereotaxic frame where isofluorane (0.9-1.5%) was continuously delivered for the duration of the surgery. Mice were then injected with buprenorphine (0.1 mg/kg, s.c.) and carpfrofen (5 mg/kg, s.c.) and bupivicane (0.25mg, under the skin of the scalp). The skin was then covered with betadine before an incision was made with a scalpel. The fascia was removed, and the skull dried with ethanol. The bregma and lamda sutures were identified and the skull was levelled using these landmarks. A craniotomy was drilled at the injection coordinates and a pulled glass pipette lowered to the injection site. An injection was made at a speed of 100 nl/min from an injection system (Nanoject III, Drummond). The injection pipette was left in place for 10 minutes following the injection then slowly retracted from the brain. In the case of bilateral injections, this process was then repeated on the other side. The skin was then closed by suture and the mouse transferred to a heated recovery cage.

For optogenetic experiments the virus injections were performed as described above. Once the injection pipette was removed the skull was scored with a scalpel blade and a fiber implant composed of a ferrule (CFLC230, Thorlabs) and an optic fiber (FT200EMT, Thorlabs) held in place with epoxy (F112, Thorlabs) inserted into the brain 200 μm dorsal to the injection site. The first fiber was glued in place while the second fiber was inserted. Once both fibers were in place, the skull was covered with dental cement (C&B Metabond) then a second layer of acrylic (Jet). After the skull cap was dried mice were transferred to a heated recovery cage. Coordinates (in mm) were as follows; iRO: 6.35 posterior to bregma, 5.4 ventral to skull surface, 1.2 lateral to midline; pBC: 6.73 posterior to bregma, 5.77 ventral to skull surface,1.3 lateral to midline.

Mice were given 6 weeks between injection/implantation surgery and being used for experiments. ReaChR mice were implanted as described above. For optogenetic experiments bilateral fibers were connected to a split-patch cord (SBP(2)_200/220/900-0.37_m_FCM-2xZF1.25, Doric Lenses) and light was delivered from a laser (MBL-III-473, Opto Engine LLC) controlled by a TTL pulse generator (OTPG_4, Doric Lenses). Mice were placed in the plethysmography chamber with the microphone attached to simultaneously record breathing and sound along with the laser pulse commands. All three data streams were acquired through a DAQ board and written to disk for offline analysis. Sound was acquired at 250 kHz, airflow at 1 kHz and laser pulse commands at 1 kHz. After a 20-minute habituation period, laser pulses were delivered at frequencies of 5, 10, 20, and 50 Hz with pulse widths of 10, 25 or 50 ms for durations of 1 or 3 seconds. Laser power was adjusted to deliver ~20 mW of light at the patch cord tip although attenuation of light by the implanted fiber (determined post-hoc) was variable (12-21 mW). Each frequency/pulse width/duration combination was delivered 5 times with 7-9 seconds between presentations and a 30s delay before the next stimulus was delivered.

Recordings were manually inspected for USVs during the laser epoch and recordings containing USVs were then run through VocalMat to find time stamps and to categorize each USV by type. Matlab code was then used to quantify the correlation coefficients of optogenetically evoked USVs and the underlying airflow as described above. To analyze the breath statistics of optogenetically evoked breathing, the trial with stimulation parameters: 10 Hz, 25ms pulse width, 3s duration was run through a code to extract breath statistics (Pif, Pef, Instantaneous Frequency) from the 30s period prior to stimulation and from the 5 laser epochs.

### Histology

More than 6 weeks following viral injection or the completion of optogenetic testing mice were deeply anaesthetized with isofluorane and transcardially perfused with 0.1M phosphate buffered saline (PBS) then PBS containing 4% paraformaldehyde (PFA). Brains were dissected from the fixed mice and refrigerated in 4% PFA overnight then cryoprotected in 30% sucrose in PBS. Brains were sectioned to 30 μm coronal on a freezing microtome. Sections were washed 3 times for 5 min in PBS before being incubated in blocking solution (PBS, 5% normal donkey serum, 0.3% Triton-X100) for 2 hours. Sections were then incubated overnight in primary antibodies (Chicken anti-GFP, 1:1000, Aves; Goat anti-ChAT, 1:500, Millipore) diluted in a carrier solution (PBS, 1% normal donkey serum, 0.3% Triton-X100). Following incubation sections were washed with PBS 5 times for 5 minutes then incubated in secondary antibodies (Donkey anti-Chicken 488, Donkey anti-Goat 546, Donkey anti-Goat 647) diluted 1:500 in a carrier solution (PBS, 0.3% Triton-X100) for 2 hours at room temperature. After secondary incubation, sections were washed with PBS 5 times for 5 minutes then mounted onto glass slides and cover-slipped with mounting media (Prolong Gold, Invitrogen) and 1 μg/ml DAPI.

## Quantification and statistical analysis

### Statistics

Data from Matlab was imported to Prism 9 (GraphPad) for statistical analysis. For all statistical analysis except figure 4 D–G the mouse was used as the experimental unit. Data were assumed to be normally distributed and of equal variance and parametric tests were used. For data with one discrete variable and measurements made from the same animal (Figure 1 D, E) paired t-test was used. For data with two variables one or both of which had more than two factors (Figure 1C, 3G, 4C,S2I,J) two-way ANOVA was used with Sidak’s post-hoc test for multiple comparisons. To compare pitch-airflow correlations of endogenous and optically-evoked USVs (Figure 4 D–G) each USV was treated as the experimental unit since the vocal repertoire across animals was similar (Figure 1F) and simply taking a mean from each animal would under-represent the complexity of the data. For comparison of correlation coeffecients between optically evoked and endogenous USVs, two way ANOVA with Sidak’s post-hoc test for two way comparisons was used. P-values below 0.05 were considered statistically significant.

## Supplementary Material

1

## Figures and Tables

**Table:** Resource Table

Abbreviation	Full Name	Identifier	Supplier	Reference
Mice				
*Vglut2* ^FlpO^	Slc17a6-IRES2-FlpO-D knock-in	030212	The Jackson Laboratory	Daigle *et al* 2018
*Penk* ^Cre^	Penk-IRES2-Cre	025112	The Jackson Laboratory	Tasic *et al* 2018
LSL-FSF-ReaChR	R26 LSL FSF ReaChR-mCitrine	024846	The Jackson Laboratory	Hooks *et al* 2015
*Tac1* ^Cre^	Tac1-IRES2-Cre-D	021877	The Jackson Laboratory	Harris *et al* 2014
*Oprm1* ^Cre^	Oprm1^Cre:GFP^ KIKO	035574	The Jackson Laboratory	Liu *et al* 2022
*Vgat* ^Cre^	Vgat-ires-cre knock-in	028862	The Jackson Laboratory	Vong *et al* 2011
Ai65	Ai65(RCFL-tdT)-D	021875	The Jackson Laboratory	Madisen *et al* 2015
Virues				
AAV-C_ON_F_ON_-ChR2-Eyfp	AAV5-hSyn-Con/Fon-hChR2(H134R)-EYFP	55645-AAV5	Addgene	Fenno *et al* 2014
AAV-DIO-ChR2	AAV5-EF1a-DIO-hChR2(H134R)-EYFP-WPRE-HGHpA	20298-AAV5	Addgene	
AAVrg-DIO-ChR2-EYFP	AAVrg-EF1a-DIO-hChR2(H134R)-EYFP-WPRE-HGHpA	20298-AAVrg	Addgene	
Antibodies				
Chicken anti-GFP		GFP-1020	Aves	
Goat anti-ChAT		AB144p	Millipore	
Donkey anti-Chicken 488		A78948	Invitrogen	
Donkey anti-Goat 546		A11056	Invitrogen	
Donkey anti-Goat 647		A21447	Invitrogen	
Software and algorithms				
Matlab		Matlab 2022b	Mathworks	
VocalMat		https://github.com/ahof1704/VocalMat		Fonseca *et al* 2021
USVseg		https://github.com/rtachilab/usvseg		Tachibana *et al* 2020
Bespoke code		https://github.com/YackleLab		This paper, Bachmutsky *et al* 2020, Wei *et al* 2022
Prism 9			GraphPad	

## Data Availability

All data collected in this study and code use for analysis are available upon request from the corresponding author.

## References

[R1] Anderson T.M., Garcia A.J., Baertsch N.A., Pollak J., Bloom J.C., Wei A.D., Rai K.G., Ramirez J.M. (2016). A novel excitatory network for the control of breathing. Nature 536, 76–80. 27462817 10.1038/nature18944 PMC5479418

[R2] Bachmutsky I., Wei X.P., Kish E., Yackle K. (2020) Opioids depress breathing through two small brainstem sites. eLife 10.7554/eLife.52694 PMC7077984 32073401

[R3] Berke G.S., and Long J.L. (2009). Functions of the larynx and production of sounds. Handbook of mammalian vocalization – an integrative neuroscience approach, Chapter 10.1.

[R4] Castellucci G.A., Calbick D., and McCormick D. (2018) The temportal organization of mouse ultrasonic vocalizations. PloS One 13(10):e0199929. 30376572 10.1371/journal.pone.0199929 PMC6207298

[R5] Chagnaud B.P., Baker R., and Bass A.H. (2011). Vocalization frequency and duration are coded in separate hindbrain nuclei. Nature communications 2, 346–11. 10.1038/ncomms1349 PMC3166519 21673667

[R6] Chen J., Markowitz J.E., Lilascharoen V., Taylor S., Sheurpukdi P., Keller J.A., Jensen J.R., Lim B.K., Datta S.R., and Stowers L. (2021). Flexible scaling and persistence of social vocal communication. Nature 593, 108–113. 33790464 10.1038/s41586-021-03403-8 PMC9153763

[R7] Daigle T.L. (2018) A suite of transgenic driver and reporter mouse lines with enhanced brain-cell-type targeting and functionality. Cell 174, 465–480 30007418 10.1016/j.cell.2018.06.035 PMC6086366

[R8] Dichter B.K., Breshears J.D., Leonard M.K., and Chang E.F. (2018). The control of vocal pitch in human laryngeal motor cortex. Cell 174, 21–31. 29958109 10.1016/j.cell.2018.05.016 PMC6084806

[R9] Finck C., and Lejeune L. (2009). Structure and oscillatory function of the vocal folds. Handbook of mammalian vocalization – an integrative neuroscience approach, Chapter 10.2.

[R10] Fenno L.E. (2014). Targeting cells with single vectors using multiple-feature Boolean logic. Nature Methods 11(7), 763–772. 24908100 10.1038/nmeth.2996 PMC4085277

[R11] Fonseca A.H., Santana G.M., Bosque Ortiz G.M., Bampi S., and Dietrich M. (2021) Analysis of ultrasonic vocalizations from mice using computer vision and machine learning. Elife 10:e59161. 33787490 10.7554/eLife.59161 PMC8057810

[R12] Goller F., and Cooper B.G. (2004). Peripheral motor dynamics of song production in the zebra finch. Ann. N. York Acad. Sci. 1016, 130–152. 10.1196/annals.1298.009 15313773

[R13] Grimsley J.M.S., Monaghan J.J.M., and Wenstrup J.J. (2011). Development of social vocalizations in mice. Plos One 6, e17460. 21408007 10.1371/journal.pone.0017460 PMC3052362

[R14] Hage S.R. (2009). Neuronal networks involved in the generation of vocalization. Handbook of behavioral neuroscience 19, 339–349.

[R15] Hage S.R. (2009). Localization of the central pattern generator for vocalization. Handbook of behavioral neuroscience 19, 329–337.

[R16] Harris J.A. (2014). Anatomical characterization of cre driver mice for neural circuit mapping and manipulation. Frontiers in Neural Circuits 8, 76. 25071457 10.3389/fncir.2014.00076 PMC4091307

[R17] Herbst C.T. (2016). Vertebrate Sound Production and Acoustic Communication. Springer handbook of auditory research, 159–189.

[R18] Hooks B.M., Lin J.Y., Guo C., Svoboda K. (2015). Dual-channel circuit mapping reveals sensorimotor convergence in the primary motor cortex. Journal of Neuroscience 35(10), 4418–4426. 25762684 10.1523/JNEUROSCI.3741-14.2015 PMC4355205

[R19] Huff A., Karlen-Amarante M., Oliveira L.M., Ramirez J.M. (2023) Role of the postinspiratory complex in regulating swallow-breathing coordination and other laryngeal behaviors. Elife 12:e86103. 37272425 10.7554/eLife.86103 PMC10264072

[R20] Johnson A.M., Ciucci M.R., Russell J.A., Hammer M.J., and Connor N.P. (2010). Ultrasonic output from the excised rat larynx. The journal of the acoustical society of america 128, EL75–EL79. 20707418 10.1121/1.3462234 PMC2924901

[R21] Jürgens U. (2002). Neural pathways underlying vocal control. Neuroscience and biobehavioral reviews 26, 235–258. 11856561 10.1016/s0149-7634(01)00068-9

[R22] Kelley D.B., Ballagh I.H., Barkan C.L., Bendesky A., Elliott T.M., Evans B.J., Hall I.C., Kwon Y.M., Kwong-Brown U., Leininger E.C., (2020). Generation, coordination, and evolution of neural circuits for vocal communication. J Neurosci 40, 22–36. 31896561 10.1523/JNEUROSCI.0736-19.2019 PMC6939475

[R23] Kelm-Nelson C.A., Lenell C., Johnson A.M., and Ciucci M.R. (2018). Laryngeal Activity for Production of Ultrasonic Vocalizations in Rats. Handbook of mammalian vocalization – an integrative neuroscience approach, Chapter 4.

[R24] Laplagne D.A. (2018). Interplay Between Mammalian Ultrasonic Vocalizations and Respiration. Neuronal networks involved in the generation of vocalization, Chapter 6.

[R25] Liu S., Ye M., Pao G.M., Song S.M., Jhang J., Jiang H., Kim J-H., Kang S.J., Kim D-I., Han S. (2022). Divergent brainstem opioidergic pathways that coordinate breathing with pain and emotions. Neuron 110(5), 857–873. 34921781 10.1016/j.neuron.2021.11.029 PMC8897232

[R26] Mahrt E., Agarwal A., Perkel D., Portfors C., and Elemans C.P.H. (2016). Mice produce ultrasonic vocalizations by intra-laryngeal planar impinging jets. Current biology 26, 1–2. 27728788 10.1016/j.cub.2016.08.032

[R27] Madisen L., (2015). Transgenic mice for intersectional targeting of neural sensors and effectors with high specificity and performance. Neuron 85(5), 942–958. 25741722 10.1016/j.neuron.2015.02.022 PMC4365051

[R28] Marder E., and Bucher D. (2001). Central pattern generators and the control of rhythmic movements. Curr Biol 11, R986–R996. 11728329 10.1016/s0960-9822(01)00581-4

[R29] Marder E. (2012). Neuromodulation of neuronal circuits: back to the future. Neuron 76, 1–11. 23040802 10.1016/j.neuron.2012.09.010 PMC3482119

[R30] Michael V., Goffinet J., Pearson J., Wang F., Tschida K., and Mooney R. (2020). Circuit and synaptic organization of forebrain-to-midbrain pathways that promote and suppress vocalization. Elife 9, e63493. 33372655 10.7554/eLife.63493 PMC7793624

[R31] Plummer E.M., and Goller F. (2008). Singing with reduced air sac volume causes uniform decrease in airflow and sound amplitude in the zebra finch. J. exp. biol. 211, 66–78. 18083734 10.1242/jeb.011908

[R32] Poeppel D., and Assaneo M.F. (2020). Speech rhythms and their neural foundations. Nature Reviews Neuroscience, 1–13. 32376899 10.1038/s41583-020-0304-4

[R33] Prieto P. (2015). Intonational meaning. Wiley Interdiscip Rev Cognitive Sci 6, 371–381. 10.1002/wcs.1352 26263426

[R34] Riede T. (2011). Subglottal pressure, tracheal airflow, and intrinsic laryngeal muscle activity during rat ultrasound vocalization. Journal of neurophysiology 106, 2580–2592. 21832032 10.1152/jn.00478.2011 PMC3214115

[R35] Riede T. (2013). Stereotypic Laryngeal and Respiratory Motor Patterns Generate Different Call Types in Rat Ultrasound Vocalization. Journal of experimental zoology part A: ecological genetics and physiology 319, 213–224. 23423862 10.1002/jez.1785 PMC3926509

[R36] Riede T., Borgard H.L., and Pasch B. (2017). Laryngeal airway reconstruction indicates that rodent ultrasonic vocalizations are produced by an edge-tone mechanism. Royal society open science 4, 170976. 29291091 10.1098/rsos.170976 PMC5717665

[R37] Schmidt M.F., and Wild J.M. (2014). Chapter 15 The respiratory-vocal system of songbirds Anatomy, physiology, and neural control. Prog. brain res. 212, 297–335. 25194204 10.1016/B978-0-444-63488-7.00015-X PMC4532670

[R38] Silva A.B., Liu J.R., Zhao L., Levy D.F., Scott T.L., and Chang E.F. (2022). A neurosurgical functional dissection of the middle precentral gyrus during speech production. J Neurosci Official J Soc Neurosci 42, 8416–8426. 10.1523/JNEUROSCI.1614-22.2022 PMC9665919 36351829

[R39] Sirotin Y.B., Costa M.E., Laplagne D.A. (2014). Rodent ultrasonic vocalizations are bound to active sniffing behavior. Frontiers in Beh. Neurosci. 8:399. 10.3389/fnbeh.2014.00399 PMC4235378 25477796

[R40] Smith J.C., Ellenberger H.H., Ballanyi K., Richter D.W., and Feldman J.L. (1991). Pre-Bötzinger complex: a brainstem region that may generate respiratory rhythm in mammals. Science 254, 726–729. 1683005 10.1126/science.1683005 PMC3209964

[R41] Suthers R.A., Goller F., and Wild J.M. (2002). Somatosensory feedback modulates the respiratory motor program of crystallized birdsong. Proc. natl. acad. sci. 99, 5680–5685. 11943843 10.1073/pnas.042103199 PMC122831

[R42] Tachibana R.O., Kanno K., Okabe S., Kobayasi K.I., Okanoya K. (2020) USVSEG: A robust method for segmentation of ultrasonic vocalizations in rodents. PLoS One10.1371/journal.pone.0228907 PMC7010259 32040540

[R43] Tasic B. (2018). Shared and distinct transcriptomic cell types across neocortical areas. Nature 563, 72–78. 30382198 10.1038/s41586-018-0654-5 PMC6456269

[R44] Tschida K., Michael V., Takato J., Han B.-X., Zhao S., Sakurai K., Mooney R., and Wang F. (2019). A specialized neural circuit gates social vocalizations in the mouse. Neuron, 103, 459–472. 31204083 10.1016/j.neuron.2019.05.025 PMC6687542

[R45] Vong L., Ye C., Yang Z., Choi B., Chua S. Jr, Lowell B.B. (2011). Leptin action on GABAergic neurons prevents obesity and reduces inhibitory tone to POMC neurons. Neuron 71, 142–54. 21745644 10.1016/j.neuron.2011.05.028 PMC3134797

[R46] Wei X.P., Collie M., Dempsey B., Fortin G., and Yackle K. (2022). A novel reticular node in the brainstem synchronizes neonatal mouse crying with breathing. Neuron 110, 644–657. 34998469 10.1016/j.neuron.2021.12.014 PMC8857054

[R47] Yackle K. (2023). Transformation of our understanding of breathing control by molecular tools. Annu rev physiol 85, 93–113. 36323001 10.1146/annurev-physiol-021522-094142 PMC9918693

[R48] Zhang Y.S., and Ghazanfar A.A. (2020). A Hierarchy of autonomous systems for vocal production. Trends in neurosciences 43, 1–12. 31955902 10.1016/j.tins.2019.12.006 PMC7213988

