# Supplementary material for: The breath shape controls intonation of mouse vocalizations
Source: bioRxiv. 2023 Oct 17:2023.10.16.562597. Preprint. [Version 1] doi: 10.1101/2023.10.16.562597 (PMC10614923; doi:10.1101/2023.10.16.562597)
Supplement: Supplementary file 1 [file NIHPP2023.10.16.562597v1-supplement-1.pdf]

bioRxiv preprint doi: https://doi.org/10.1101/2023.10.16.562597; this version posted October 17, 2023. The copyright holder for this preprint (which was not certified by peer review) is the author/funder, who has granted bioRxiv a license to display the preprint in perpetuity. It is made available under aCC-BY 4.0 International license.

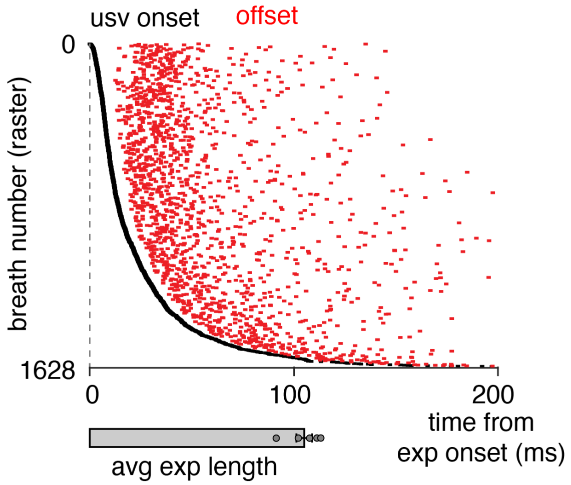

Figure Supplemental 1. USV onset and offset during expiration. Left, raster plot of USV onset and offset times (ms) aligned to the beginning of expiration (onset, black and offset, red) for 1850 events. Below, the average expiratory length for n=6 animals. Right, histogram of the onset for each vocalization during a normalized expiratory duration. Note, while onset is biased to early expiration, vocalizations can begin throughout and even in late expiration.

bioRxiv preprint doi: https://doi.org/10.1101/2023.10.16.562597; this version posted October 17, 2023. The copyright holder for this preprint (which was not certified by peer review) is the author/funder, who has granted bioRxiv a license to display the preprint in perpetuity. It is made available under aCC-BY 4.0 International license.

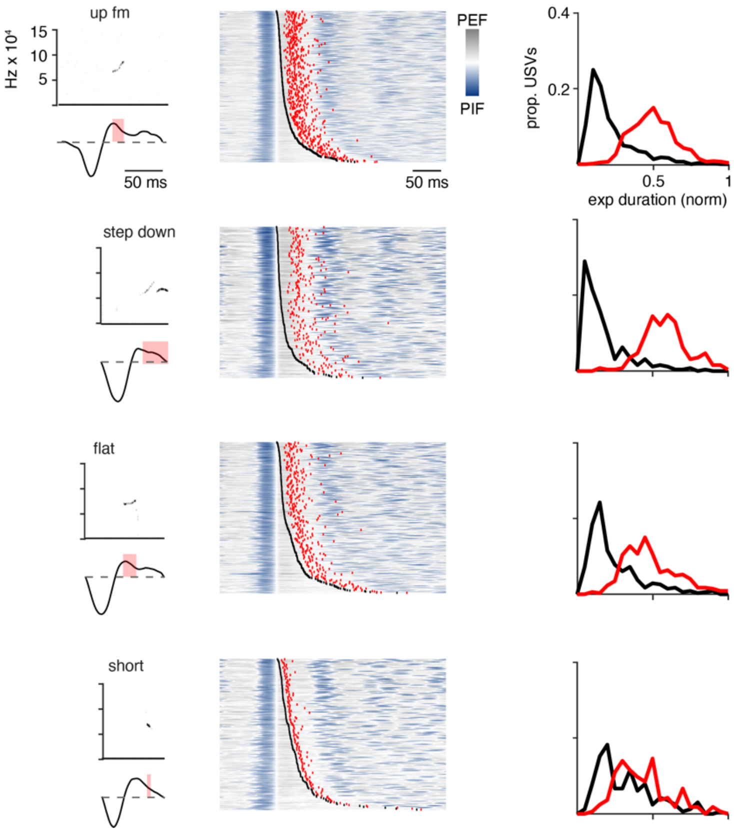

Figure Supplemental 2. Representative example of the most common USV types and the onset and offset times during expiration. Representative examples for each if the most common USV types and the representation of the onset and offset as Fig. S1.

bioRxiv preprint doi: https://doi.org/10.1101/2023.10.16.562597; this version posted October 17, 2023. The copyright holder for this preprint (which was not certified by peer review) is the author/funder, who has granted bioRxiv a license to display the preprint in perpetuity. It is made available under aCC-BY 4.0 International license.

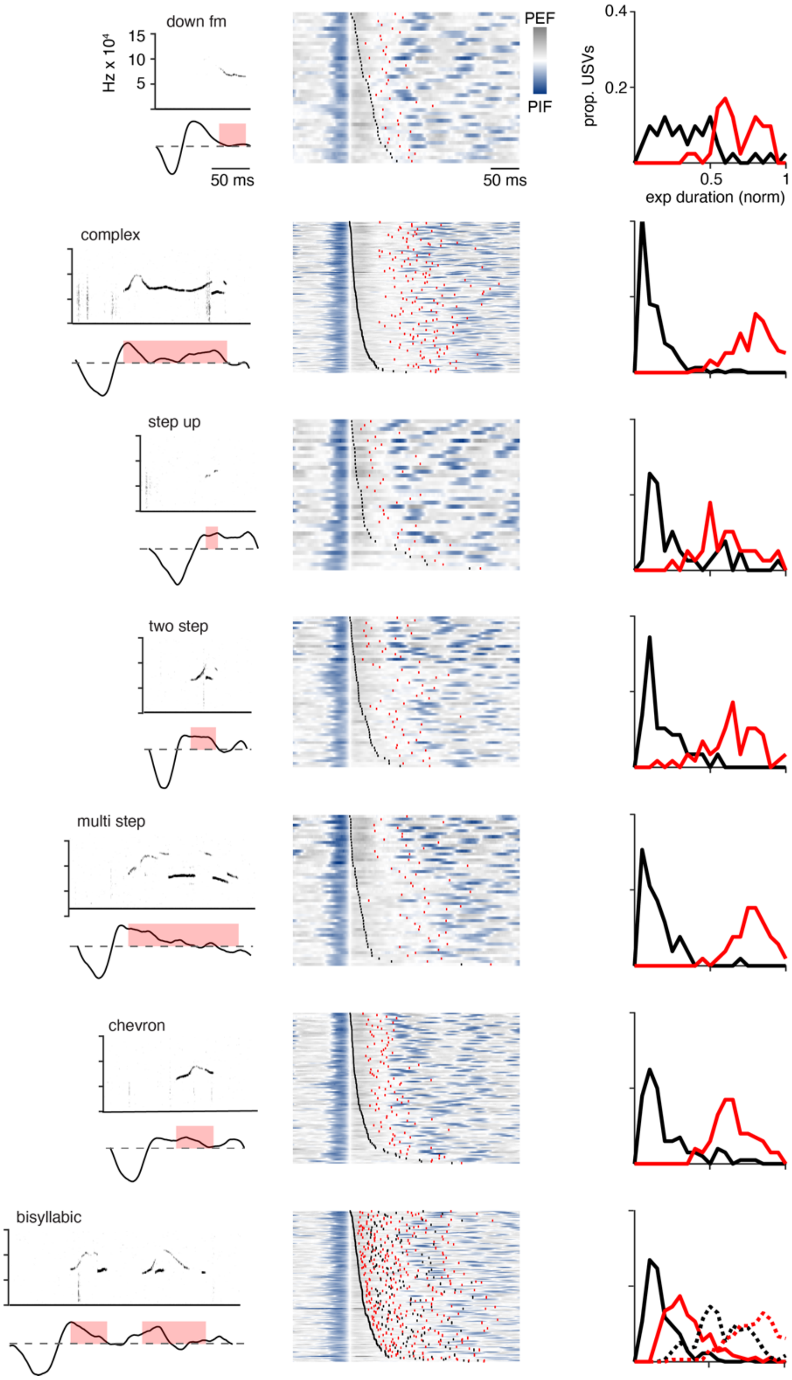

Figure Supplemental 3. Representative example of the many USV types and the onset and offset times during expiration. Representative examples for the remaining USV types and the representation of the onset and offset as Fig. S1. Note, more complex vocalizations have onset and offset times that occur later in expiration.

bioRxiv preprint doi: https://doi.org/10.1101/2023.10.16.562597; this version posted October 17, 2023. The copyright holder for this preprint (which was not certified by peer review) is the author/funder, who has granted bioRxiv a license to display the preprint in perpetuity. It is made available under aCC-BY 4.0 International license.

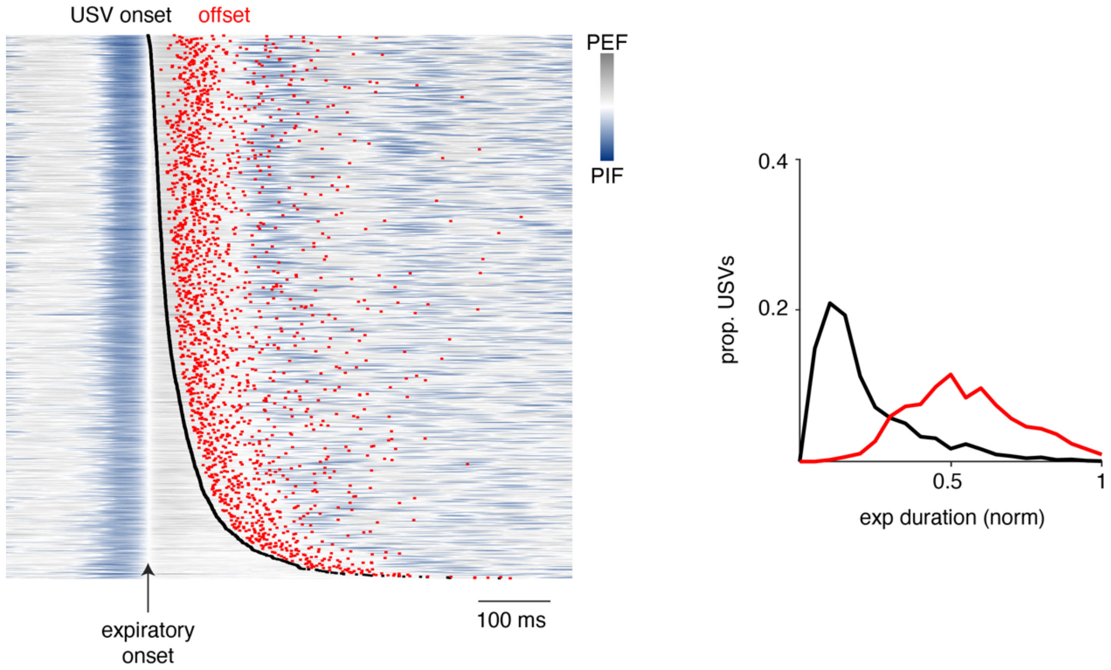

Figure Supplemental 4. Raster plot of USV on- and offset plotted upon the breathing rhythm. Raster plot of 1850 USVs aligned by the beginning of expiration with the sound onset and offset annotated by dots. The breath airflow is represented the gradient from blue to gray, where inspiration is blue, and expiration is gray. Note that breaths after ~1200 have late onset during expiration and delay the onset of the subsequent inspiration.

bioRxiv preprint doi: https://doi.org/10.1101/2023.10.16.562597; this version posted October 17, 2023. The copyright holder for this preprint (which was not certified by peer review) is the author/funder, who has granted bioRxiv a license to display the preprint in perpetuity. It is made available under aCC-BY 4.0 International license.

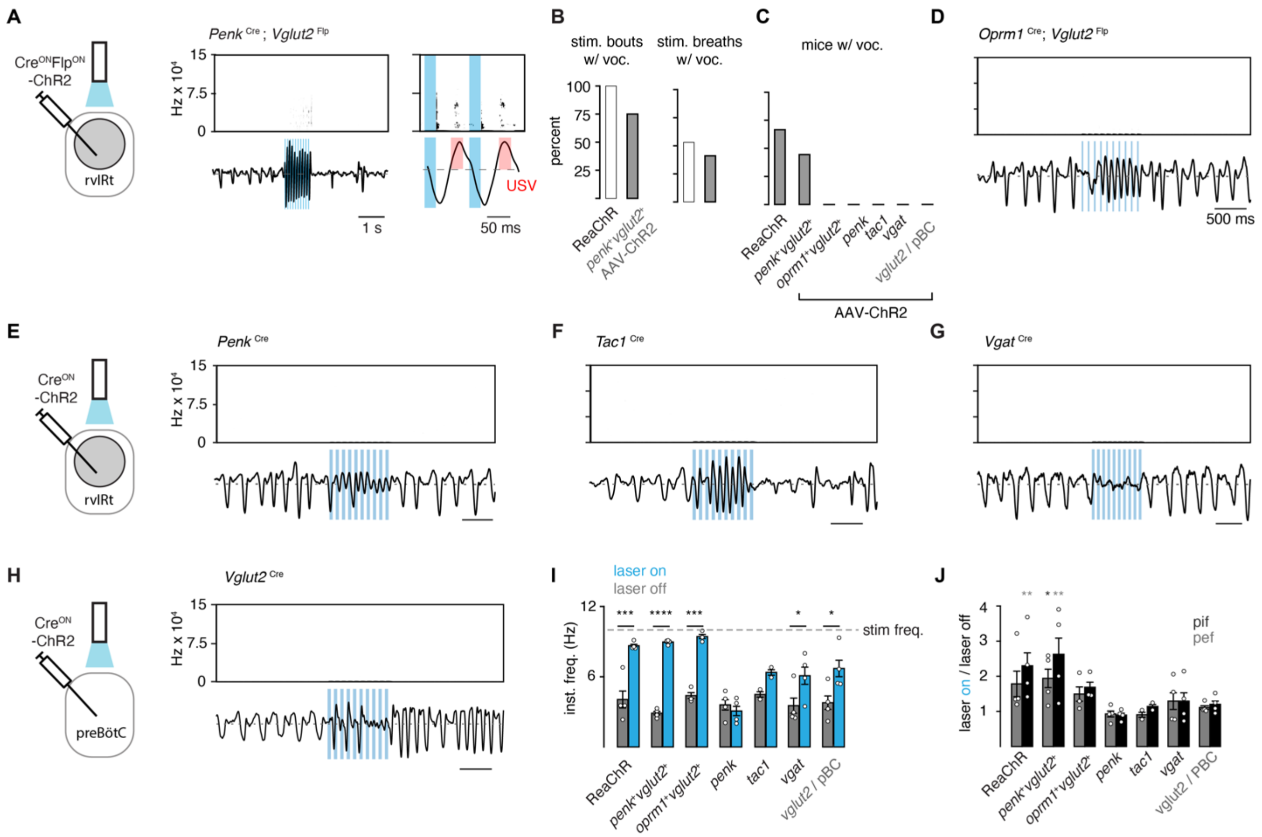

Figure Supplemental 6. Optogenetic modulation of breathing and USVs for different molecularly defined cell types in the iRO anatomical region. A, Representative example of the change in breathing and ultrasonic vocalizations during a single light stimulation bout (blue box, 10 Hz) in *Penk*-Cre;*Vglut2*-Flp mice stereotaxically injected with AAV Cre^{ON}Flp^{ON}-Channel Rhodopsin2::YFP (ChR2) in the iRO (gray circle). Breathing rate is entrained by light and the amplitude is increased. USVs occur at the peak of expiration. rvIRt, rostral ventral Intermediate Reticular tract. **B**, Percent of stimulation bouts and breaths within each bout that contain USVs or broad band vocalizations in *Penk*-Cre;*Vglut2*-Flp;ReaChR and *Penk*-Cre;*Vglut2*-Flp virally injected mice. **C**, Percent of mice with vocalizations for each tested genotype and injection site.

bioRxiv preprint doi: https://doi.org/10.1101/2023.10.16.562597; this version posted October 17, 2023. The copyright holder for this preprint (which was not certified by peer review) is the author/funder, who has granted bioRxiv a license to display the preprint in perpetuity. It is made available under aCC-BY 4.0 International license.

ReaChR with iRO optic fiber implantation, n=6. iRO stereotaxic viral injection: *Penk*-Cre;*Vglut2*-Flp, n=9; *Oprm1*-Cre;*Vglut2*-Flp, n=4; *Penk*-Cre, n=4; *Tac1*-Cre, n=5, *Vgat*-Cre, n=4. PreBötC stereotaxic viral injection: *Vglut2*-Cre, n=4. **D-H**, Representative examples of stimulation bouts for each genotype with rvIRt or preBötC viral injection. **I**, Bar graph of average ± standard deviation and average for each animal (circle) for the instantaneous breathing frequency before and during the optogenetic laser pulse (10 Hz). * $p<0.05$; *** $p<0.001$; **** $p<0.0001$; two-way ANOVA with Sidak's post-hoc test. Genotypes and injection sites as in **C-H**. **J**, Bar graph of average ± standard deviation and average for each animal (circle) for the ratio of the peak inspirator flow (pif, black) and peak expiratory flow (pef, gray) for optogenetically stimulated breaths versus nearby unstimulated breaths for each genotype. * $p<0.05$; ** $p<0.01$; two-way ANOVA with Sidak's post-hoc test. Genotypes and injection sites as in **C-H.**
